# Dysregulated lipid metabolism in TMZ-resistant glioblastoma: pathways, proteins, metabolites and therapeutic opportunities

**DOI:** 10.1186/s12944-023-01881-5

**Published:** 2023-08-03

**Authors:** Tzu-Jen Kao, Chien-Liang Lin, Wen-Bin Yang, Hao-Yi Li, Tsung-I Hsu

**Affiliations:** 1 Ph.D. Program in Medical Neuroscience, College of Medical Science and Technology, Taipei Medical University and National Health Research Institutes, Taipei, 110 Taiwan; 2 International Master Program in Medical Neuroscience, College of Medical Science and Technology, Taipei Medical University, Taipei, 110 Taiwan; 3 TMU Research Center of Neuroscience, Taipei Medical University, Taipei, 110 Taiwan; 4 Chi Mei Medical Center, Tainan, 71004 Taiwan; 5 Department of Biochemistry, Ludwig-Maximilians-University, Munich, 81377 Germany; 6 Gene Center, Ludwig-Maximilians-University, Munich, 81377 Germany; 7 TMU Research Center of Cancer Translational Medicine, Taipei, 110 Taiwan; 8 Ph.D. Program in Drug Discovery and Development Industry, College of Pharmacy, Taipei Medical University, Taipei, 110 Taiwan

## Abstract

Glioblastoma (GBM) is a highly aggressive and lethal brain tumor with limited treatment options, such as the chemotherapeutic agent, temozolomide (TMZ). However, many GBM tumors develop resistance to TMZ, which is a major obstacle to effective therapy. Recently, dysregulated lipid metabolism has emerged as an important factor contributing to TMZ resistance in GBM. The dysregulation of lipid metabolism is a hallmark of cancer and alterations in lipid metabolism have been linked to multiple aspects of tumor biology, including proliferation, migration, and resistance to therapy. In this review, we aimed to summarize current knowledge on lipid metabolism in TMZ-resistant GBM, including key metabolites and proteins involved in lipid synthesis, uptake, and utilization, and recent advances in the application of metabolomics to study lipid metabolism in GBM. We also discussed the potential of lipid metabolism as a target for novel therapeutic interventions. Finally, we highlighted the challenges and opportunities associated with developing these interventions for clinical use, and the need for further research to fully understand the role of lipid metabolism in TMZ resistance in GBM. Our review suggests that targeting dysregulated lipid metabolism may be a promising approach to overcome TMZ resistance and improve outcomes in patients with GBM.

## Introduction

Glioblastoma (GBM) is the most common and aggressive primary brain tumor in adults, with limited treatment options and a poor prognosis [1]. Pathogenesis of GBM involves a complex interplay of genetic, epigenetic, and environmental factors, leading to uncontrolled cell division, immune evasion, angiogenesis, and invasion of surrounding brain tissue. Current standard-of-care treatment protocol for newly diagnosed GBM includes maximal safe surgical resection followed by radiotherapy and adjuvant chemotherapy with temozolomide (TMZ). Despite this aggressive multimodal treatment, prognosis remains dismal with a median survival of about 14–16 months. However, GBM tumors are notoriously heterogeneous which complicates treatment and often leads to therapeutic resistance. Therefore, even with current treatment regimens, most patients experience tumor recurrence within a year of initial diagnosis. The dysregulation of lipid metabolism is now recognized as a hallmark of cancers, including GBM, and has been linked to multiple aspects of tumor biology, including proliferation, migration, and resistance to therapy [2]. In this review, we aimed to provide an overview of current knowledge on lipid metabolism in GBM, including the key pathways and proteins involved, and recent advances in the application of metabolomics to study lipid metabolism in GBM. We also discussed the potential of lipid metabolism as a target for novel therapeutic interventions and the challenges and opportunities associated with developing these interventions for clinical use.

## Obstacle to clinical GBM treatment

GBM is a highly aggressive brain cancer with poor prognosis. Despite advances in medical technology and the development of new therapies, the survival rate of patients with GBM remains low. One of the main obstacles to clinical GBM treatment is treatment resistance [3]. GBM is a heterogeneous disease that involves different cell types that respond differently to therapy. GBM cells can also develop resistance to treatment over time, making it difficult to achieve long-term remission. Resistance mechanisms in GBM are complex and multifactorial but can be broadly classified into intrinsic and extrinsic factors [4].

Intrinsic resistance refers to genetic and epigenetic changes that occur within tumor cells. Mutations in genes involved in DNA repair, cell cycle regulation, and apoptosis can confer resistance to chemotherapy and radiation therapy. Epigenetic changes, such as alterations in DNA methylation and histone modifications, can also contribute to treatment resistance [5]. Extrinsic resistance refers to factors outside tumor cells that affect the response to therapy. Particularly, the blood-brain barrier (BBB) is a physiological barrier that protects the brain from foreign substances, including chemotherapy drugs. The BBB can limit the delivery of therapeutic agents to tumors, reducing their efficacy [6]. The tumor microenvironment can also influence treatment response. In particular, the presence of immune cells and other stromal cells can create a protective shield around the tumor, making it difficult for chemotherapy and radiation therapy to reach the cancer cells [7].

Another obstacle to the clinical treatment of GBM is the lack of effective targeted therapies. Targeted therapies specifically target molecular pathways involved in cancer cell growth and survival. However, the genetic heterogeneity of GBM makes it difficult to identify targetable pathways that are common to all patients. Furthermore, even when potential targets are identified, it can be challenging to develop drugs that effectively target them in the brain due to the BBB [8]. The aggressive nature of GBM poses challenges to clinical management. GBM tumors are known for their rapid growth and invasion of surrounding brain tissue, which can make it difficult to achieve complete surgical resection. Additionally, GBM is prone to recurrence, even after aggressive treatment. Recurrent tumors often have a different genetic profile from the original tumor, which can make them more resistant to therapy [9].

Treatment resistance is a major obstacle to the clinical treatment of GBM. The complex and multifactorial nature of resistance mechanisms, combined with the lack of effective targeted therapies, makes it difficult to achieve long-term remission in patients with GBM. Overcoming these obstacles will require a better understanding of the biology of GBM, development of novel therapies, and innovative approaches for drug delivery and clinical management.

## Alteration of metabolism in drug-resistant glioblastoma

One of the key factors contributing to treatment resistance is metabolic alterations. GBM cells have a high metabolic rate, which allows them to adapt and survive in a hostile tumor microenvironment [10]. Glucose metabolism is the central pathway providing energy for cell growth and proliferation. GBM cells have a high demand for glucose and often rely on glycolysis even in the presence of oxygen (aerobic glycolysis or the Warburg effect). This metabolic adaptation allows GBM cells to produce ATP more quickly and supports the biosynthesis of macromolecules, such as nucleotides and lipids. In drug-resistant GBM, alterations in glucose metabolism have been observed, including the upregulation of glucose transporters, glycolytic enzymes, and lactate dehydrogenase. These changes can confer resistance to chemotherapy and radiation therapy and promote tumor growth and invasion [11]. Lipid metabolism is another key pathway altered in drug-resistant GBM. Lipids are essential for cell membrane structure, energy storage, and signaling. In GBM, alterations in lipid metabolism have been observed, including increased synthesis of fatty acids and upregulation of lipid transporters, such as CD36 and fatty acid binding protein (FABP). These changes contribute to drug resistance by promoting cell survival and increasing the availability of lipid precursors for membrane biosynthesis and energy production [12]. Metabolic alterations contribute to drug resistance in GBM. These alterations can affect several metabolic pathways, including glucose and lipid metabolisms. Understanding these metabolic alterations and developing strategies to target them may improve the efficacy of chemotherapy and radiotherapy in patients with GBM.

## Lipid metabolism for acquiring temozolomide (TMZ) resistance in glioblastoma

### Aberrant activation of lipid metabolism

Recently, alterations in lipid metabolism have been suggested to play a key role in the acquisition of TMZ resistance in GBM [2]. As shown in Table 1, multiple lipid metabolites are associated with TMZ resistance in GBM. In particular, decreased levels of sphingomyelins [13, 14], polyunsaturated fatty acids (PUFAs) [15–17], glutathione [18, 19], and taurine [20, 21] may contribute to TMZ resistance by impairing cell membrane functions and antioxidant homeostasis. Increased levels of phosphatidylcholines [22, 23], phosphatidylethanolamines [16, 23], and myo-inositol [24, 25] may be related to altered membrane composition or signaling pathways in TMZ-resistant GBM. Further research is required to fully understand the role of these metabolites in TMZ resistance and develop effective diagnostic and therapeutic strategies.

**Table 1:** Summarizing the important lipid metabolites in TMZ-resistant GBM

Metabolite	Function	Consequence in TMZ-resistant GBM
Sphingomyelins	Structural component of cell membranes	Decreased levels observed in TMZ-resistant GBM [13, 14]
Phosphatidylcholines	Structural component of cell membranes	Increased levels observed in TMZ-resistant GBM [22, 23]
Phosphatidylethanolamine	Structural component of cell membranes	Increased levels observed in TMZ-resistant GBM [16, 23, 26]
Polyunsaturated fatty acids (PUFAs)	Structural components of cell membranes and signaling molecules	Decreased levels observed in TMZ-resistant GBM [15–17]
Glutathione	Antioxidant	Decreased levels observed in TMZ-resistant GBM [18, 19]
Taurine	Antioxidant	Decreased levels observed in TMZ-resistant GBM [20, 21]
N-Acetylaspartate	Involved in energy metabolism and neurotransmission	Decreased levels observed in TMZ-resistant GBM [27–29]
Creatine	Involved in energy metabolism and brain function	Decreased levels observed in TMZ-resistant GBM [30, 31]
Myo-inositol	Involved in cell signaling and osmoregulation	Increased levels observed in TMZ-resistant GBM [24, 25]
Choline	Precursor for synthesis of phospholipids and signaling molecules	Increased levels observed in TMZ-resistant GBM [32, 33]

Lipids are important sources of energy and building blocks for cell membranes and signaling molecules. In cancer cells, including GBM cells, lipid metabolism is often altered to meet the high energy demands of rapid cell proliferation. Several studies have shown that GBM cells have a distinct lipid profile compared with normal brain tissue, including alterations in fatty acid synthesis, uptake, and utilization [12].

Alterations in lipid metabolism have been observed in TMZ-resistant GBM cells. TMZ-resistant GBM cells were found to have increased levels of long-chain fatty acids, such as palmitate and stearate, and decreased levels of PUFAs, such as arachidonic acid (AA) and docosahexaenoic acid. These changes in lipid composition are associated with the increased expression of fatty acid synthase (FASN), an enzyme involved in fatty acid synthesis, and decreased expression of delta-6-desaturase (D6D), an enzyme involved in PUFA synthesis [34]. Other studies have revealed that GBM cells with acquired TMZ resistance exhibit increased expression of fatty acid transport proteins and FABP7, which are involved in fatty acid uptake and transport. This increased expression of lipid transport proteins may contribute to the higher levels of fatty acids in TMZ-resistant GBM cells [35]. The importance of lipid metabolism in acquiring TMZ resistance in GBM is further supported by the fact that the inhibition of fatty acid synthesis or uptake can sensitize GBM cells to TMZ treatment. Inhibiting stearoyl-CoA desaturase, an enzyme involved in fatty acid metabolism, sensitizes GBM cells to radiation therapy [36]. Several mechanisms have been proposed to explain the contribution of alterations in lipid metabolism to TMZ resistance in GBM. For example, changes in lipid composition may affect membrane fluidity and protein localization, which can affect drug uptake and efflux. Additionally, alterations in lipid metabolism may affect the signaling pathways involved in cell survival and apoptosis, such as the PI3K/Akt/mTOR and NF-kB pathways [37, 38].

### Important proteins for lipid metabolism in TMZ-resistant GBM

Several proteins are important for lipid metabolism in TMZ-resistant GBM; thus, targeting these proteins may lead to new treatment strategies (Table 2). FASN is a key enzyme in de novo fatty acid synthesis and its upregulation has been implicated in the development of TMZ resistance in GBM. The inhibition of FASN was found to sensitize TMZ-resistant GBM cells to chemotherapy [39, 40]. Sterol regulatory element-binding protein (SREBP) is a transcription factor that regulates the expression of genes involved in cholesterol and fatty acid metabolism. SREBP activation promotes TMZ resistance in GBM by upregulating fatty acid synthesis [2, 41, 42]. Acetyl-CoA carboxylase (ACC) is an enzyme that catalyzes the conversion of acetyl-CoA to malonyl-CoA, which is a precursor for fatty acid synthesis. ACC inhibition has been demonstrated to reduce the proliferation and de novo lipogenesis of GBM [43]. Carnitine palmitoyltransferase 1 A (CPT1A) is an enzyme involved in fatty acid oxidation, the process by which fatty acids are broken down to produce energy. The inhibition of CPT1A has been shown to sensitize GBM cells to chemotherapy [44, 45]. Peroxisome proliferator-activated receptor gamma (PPARγ) is a transcription factor that regulates the expression of genes involved in lipid metabolism. Activation of PPARγ has been demonstrated to sensitize TMZ-resistant GBM cells to chemotherapy by promoting lipid accumulation and inducing cell death [46]. (Table 3).

**Table 2:** Summarizing the important proteins involved in lipid metabolism in TMZ-resistant GBM

Protein	Function	Role in TMZ resistance
Fatty acid synthase	Enzyme involved in de novo fatty acid synthesis	Upregulation promotes TMZ resistance; inhibition sensitizes GBM cells to chemotherapy [36]
Sterol regulatory element-binding protein	Transcription factor that regulates expression of genes involved in cholesterol and fatty acid metabolism	Activation promotes TMZ resistance by upregulating fatty acid synthesis [42]
Acetyl-CoA carboxylase	Enzyme that converts acetyl-CoA to malonyl-CoA, precursor for fatty acid synthesis	Inhibition suppresses proliferation and migration in GBM cells [43]
Carnitine palmitoyltransferase 1 A	Enzyme involved in fatty acid oxidation	Inhibition sensitizes GBM cells to chemotherapy [44, 45]
Peroxisome proliferator-activated receptor gamma	Transcription factor that regulates expression of genes involved in lipid metabolism	Activation sensitizes TMZ-resistant GBM cells to chemotherapy by promoting lipid accumulation and inducing cell death [46, 47]

**Table 3:** Summarizing the important proteins involved in steroids and arachidonate metabolism in TMZ-resistant GBM

Protein	Function	Role in TMZ resistance
Cytochrome P450 enzymes	Family of enzymes involved in metabolism of steroids, drugs, and other compounds	CYP17A1 and CYP19A1 have been implicated in TMZ resistance in GBM [48–50]
Prostaglandin E synthase	Enzyme that catalyzes conversion of prostaglandin H2 to prostaglandin E2	Upregulation observed in TMZ-resistant GBM cells [44]
5-Lipoxygenase	Enzyme that catalyzes conversion of arachidonic acid to leukotrienes	Expression shown to be upregulated in TMZ-resistant GBM cells [51]
Androgen receptor	Nuclear receptor that regulates expression of genes involved in tumor malignancy	Activation promotes TMZ resistance in GBM; inhibition sensitizes GBM cells to chemotherapy [52]

These proteins are potential targets for the development of new treatment strategies for TMZ-resistant GBM. Inhibition of fatty acid synthesis or activation of fatty acid oxidation may sensitize GBM cells to chemotherapy; however, activation of lipid accumulation or induction of cell death may also be effective. Further research is needed to fully understand the role of these proteins in TMZ resistance and develop effective targeted therapies.

Based on these findings, alterations in lipid metabolism have emerged as important factors in the acquisition of TMZ resistance in GBM. GBM cells with acquired TMZ resistance have a distinct lipid profile characterized by increased levels of long-chain fatty acids and decreased levels of PUFAs. These changes in lipid composition are associated with increased expression of fatty acid synthase and decreased expression of D6D. Inhibition of fatty acid synthesis or uptake has been shown to sensitize GBM cells to TMZ treatment. Understanding the mechanisms underlying the alterations in lipid metabolism in GBM may provide new targets for overcoming drug resistance and improving chemotherapy efficacy in patients with GBM.

### Prospective avenues for exploring metabolic pathways in GBM

To further our understanding of lipid metabolism in TMZ-resistant GBM, an in-depth analysis of the molecular heterogeneity that characterizes GBM is needed. Considering the complexities of the disease, it is necessary to dissect the consequential differences in the lipidomic profiles of different GBM subtypes and how they relate to TMZ resistance. A comprehensive examination of fatty acid composition within GBM is crucial due to its significant impact on tumor development. In particular, previous studies employing desorption electrospray ionization mass spectrometry (DESI-MS) found fatty acid signatures varying across different histological grades of brain tumors, indicating subtle differences in lipid profiles between tumors [53]. Additionally, a recent landmark study investigating the lipidome of 99 GBMs revealed substantial differences in lipid species between tumor and normal brain tissues, based on isocitrate dehydrogenase (IDH) status and tumor molecular subtype [54]. A significant observation is the enrichment of very-long-chain fatty acids (VLCFAs) and glycerophospholipids with PUFA side chains in the proneural subtype of GBM. The mesenchymal subtype, however, exhibited an increase in overall levels of glycerolipids (triacylglycerols) and a decrease in glycerophospholipids [53, 54]. These findings underscore the immense potential for lipid metabolism as a therapeutic target in GBM, warranting further investigation into the mechanistic relationship between these lipidomic changes and TMZ resistance. The other study integrates genomic, proteomic, post-translational modification, and metabolomic data from a wide array of GBMs [54]. This integrated approach provides insights into the key phosphorylation events, potential targets for GBM with EGFR-, TP53-, and RB1-alterations, and distinct global metabolic changes in IDH-mutated tumors [54].

Therefore, future exploration in this field requires an understanding of the lipidomic landscape of GBM. This involves recognizing the genetic and molecular subtypes within tumors and devising strategies to account for tumor heterogeneity. Moreover, it’s necessary to improve the precision and accuracy of mass spectrometric techniques for lipidomics. The integration of multiple omics technologies, as shown in the previous study [54], represents a promising approach to unveiling the multifaceted nature of GBM. These integrated multi-omics investigations will not only improve our understanding of lipid metabolism in GBM but will also likely lead to novel strategies to overcome TMZ resistance.

## Steroids and arachidonate metabolites in TMZ-resistant GBM

### Upregulation of steroids and arachidonate metabolites in TMZ-resistant GBM

TMZ is a chemotherapeutic drug that is commonly used to treat GBM, a highly aggressive brain tumor. However, one of the main challenges in GBM treatment is the development of TMZ resistance. Recent studies have suggested that alterations in lipid metabolism, including the generation of steroids and arachidonate metabolites, may play a role in the acquisition of TMZ resistance in GBM [44, 48, 49, 55].

**Fig. 1:**
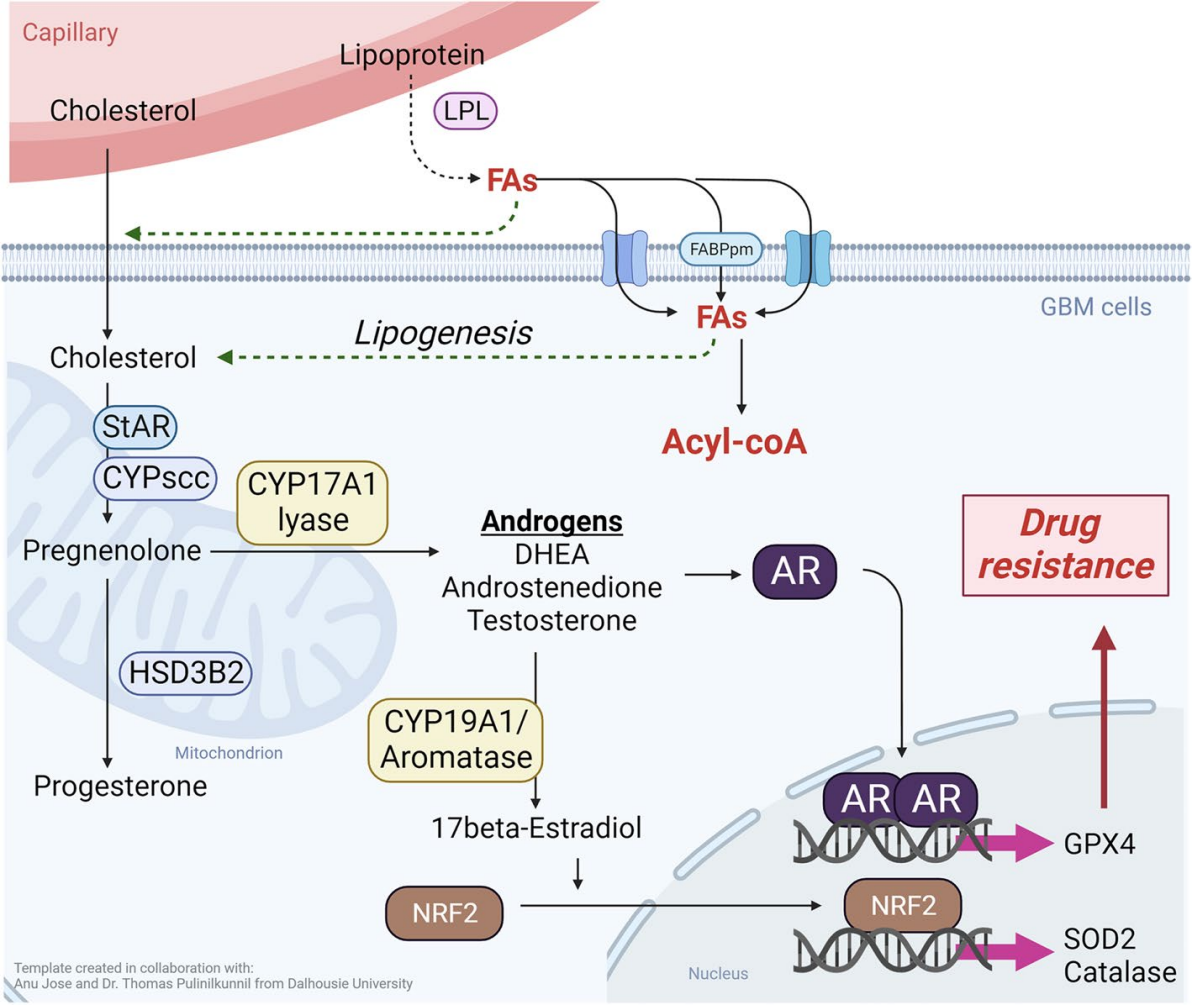
Scheme of the biosynthesis and actions of steroids in TMZ-resistant GBM. High expression levels of CYP17A1 and CYP19A1 in GBM markedly increase the tolerance of GBM cells to TMZ. By activating AR and NRF2, the expression levels of glutathione peroxidase (GPX) 4, and superoxide dismutase (SOD) 2 are remarkably increased to attenuate TMZ-induced redox damage. The figure was created with biorender.com (Account: dabiemhsu@tmu.edu.tw)

Steroids are a class of lipids that play important roles in many physiological processes including inflammation and cell proliferation. In contrast, arachidonate metabolites are derived from the breakdown of arachidonic acid, an omega-6 fatty acid involved in many cellular processes, including inflammation, and the regulation of cell growth and death [56]. Studies have shown that TMZ-resistant GBM cells have increased levels of steroids and arachidonate metabolites compared with their TMZ-sensitive counterparts [44, 48]. In addition, TMZ-resistant GBM cells increased the expression of steroidogenic enzymes, including CYP11A1, CYP17A1, and CYP19A1, which are involved in the biosynthesis of steroids, such as pregnenolone, dehydroepiandrosterone (DHEA), and 17beta-estradiol [48, 49, 57] (Fig. 1). In another study, TMZ-resistant GBM cells displayed increased expression of 5-lipoxygenase (5-LOX), which is involved in the synthesis of leukotrienes, a class of arachidonate metabolites. This increase in 5-LOX expression is associated with the increased production of leukotriene B4, a proinflammatory mediator that promotes cell survival and proliferation [51].

The importance of steroid and arachidonate metabolism in TMZ resistance is further supported by the sensitization of GBM cells to TMZ treatment via the inhibition of these pathways. Previously, the inhibition of CYP17A1 or cyclooxygenase-2 (COX-2) has been revealed to sensitize GBM cells to TMZ treatment in vitro and in vivo [44, 48]. The mechanisms underlying the role of steroid and arachidonate metabolism in TMZ resistance are not fully understood. However, these pathways may affect the expression or activity of drug efflux pumps, such as P-glycoprotein, which can pump drugs out of cells and reduce their effectiveness [58]. Alternatively, steroid and arachidonate metabolites may activate the signaling pathways involved in cell survival and proliferation, such as the PI3K/Akt and MAPK pathways [59].

Alterations in lipid metabolism, including the generation of steroids and arachidonate metabolites, have emerged as important factors in the acquisition of TMZ resistance in GBM. TMZ-resistant GBM cells have increased levels of steroids and arachidonate metabolites compared to their sensitive counterparts, and the inhibition of these pathways can sensitize GBM cells to TMZ treatment. Further research is needed to fully understand the mechanisms of lipid metabolism in TMZ resistance and develop new therapies that can target these pathways to improve the efficacy of GBM treatment.

### Important proteins for steroid and arachidonate metabolism in TMZ-resistant GBM

Several proteins involved in steroid and arachidonate metabolism are important in TMZ-resistant GBM (Table 2). Cytochrome P450 (CYP) enzymes are a family of enzymes involved in the metabolism of steroids, drugs, and other compounds. As CYP17A1 converts pregnanolone to DHEA and CYP19A1, which converts androgen to estrogen, this enzyme has been implicated in the development of TMZ resistance in GBM [48–50, 60]. Prostaglandin E synthase (PTGES) is an enzyme that catalyzes the conversion of prostaglandin (PG) H2 to PGE2, which is involved in inflammation and pain signaling. PTGES2 upregulation has also been observed in TMZ-resistant GBM cells [44]. 5-LOX catalyzes the conversion of arachidonic acid to leukotrienes, which are involved in inflammation and immune responses. Notably, 5-LOX expression is upregulated in TMZ-resistant GBM cells [51]. Androgen receptor (AR) is a nuclear receptor that regulates the expression of genes involved in metabolism, immune responses, and tumor progression. The activation of AR promotes TMZ resistance in GBM, while its inhibition sensitizes GBM cells to chemotherapy [52].

### Mechanism driven by steroids in the induction of TMZ resistance in GBM

Steroids induce resistance to TMZ therapy in GBM through multiple mechanisms. Dexamethasone, a commonly used steroid in patients with GBM, upregulates the expression of DNA repair enzymes, such as O^6^-methylguanine-DNA methyltransferase (MGMT) [61], reducing the sensitivity to TMZ therapy. By maintaining the Bax:Bcl-2 ratio, dexamethasone also protects GBM cells from TMZ-induced apoptosis [62]. Steroid receptor coactivator-1 can promote the survival of GBM stem-like cells, which might be responsible for tumor recurrence and resistance to therapy [63]. Importantly, DHEA and 17beta-estradiol levels were found to increase in TMZ-resistant GBM cells. To acquire TMZ resistance in GBM cells, DHEA and 17beta-estradiol promote DNA-repair repair and clearance of reactive oxygen species (ROS) clearance, respectively [49, 55]. The activation of AR and NRF2 by sex steroids is also involved in the development of TMZ resistance in GBM [49, 52] (Fig. 1).

In summary, steroids induce TMZ resistance in GBM through multiple mechanisms, including the upregulation of DNA repair enzymes [55], reduction of ROS accumulation [49], activation of pro-survival signaling pathways [62], promotion of stem-like cell survival [63], and modulation of the immune microenvironment [64]. Overall, these studies highlight the importance of understanding the mechanisms of steroid-induced TMZ resistance in GBM and the need for new therapeutic strategies to overcome this resistance.

### Mechanism driven by arachidonate metabolites in the induction of TMZ resistance in GBM

AA is a polyunsaturated fatty acid metabolized by COX enzymes to produce various pro-inflammatory eicosanoids, including PGE2. According to several studies, arachidonate metabolites, particularly PGE2, can induce resistance to TMZ therapy in GBM cells through multiple mechanisms. By enhancing mitochondria-mediated fatty acid beta-oxidation and TCA cycle progression, PGE2 markedly increases the tolerance of GBM cells to TMZ treatment, leading to TMZ resistance [44].

One mechanism by which PGE2 induces TMZ resistance is through the activation of the PGE2 receptor EP4 [65]. Treatment with PGE2 also increases EP2 expression, which promotes the activation of the PI3K/Akt/mTOR signaling pathway [66]. EP1/3 and EP2 inhibitors were previously found to effectively kill GBM cells and sensitize GBM cells to TMZ therapy [44, 67]. PGE2 induces TMZ resistance by activating the Wnt/β-catenin signaling pathway [68, 69]. Further, β-catenin knockdown sensitizes GBM cells to TMZ therapy [36], suggesting that the Wnt/β-catenin pathway is involved in PGE2-induced TMZ resistance. PGE2 induces TMZ resistance, partly by modulating the tumor microenvironment [70]. PGE2 promotes the recruitment of immunosuppressive myeloid-derived suppressor cells to the tumor microenvironment, which can inhibit the activity of immune cells, such as T and natural killer cells [70]. In metabolic regulation, PGE2 enhances mitochondria-mediated fatty acid oxidation, thereby increasing the tolerance of GBM cells to TMZ treatment [44].

In summary, arachidonate metabolites, including PGE2, induce TMZ resistance in GBM through multiple mechanisms, including activation of the EP2 receptor and PI3K/Akt/mTOR signaling pathway, activation of the Wnt/β-catenin signaling pathway, and modulation of the tumor microenvironment. These studies highlight the importance of understanding the mechanisms of arachidonate metabolite-induced TMZ resistance in GBM and the need for new therapeutic strategies to overcome this resistance.

### Targeting steroid and arachidonate metabolism to attenuate TMZ resistance in GBM

Targeting steroid synthesis and arachidonate metabolism may serve as promising approaches for improving the efficacy of GBM treatment. One approach for targeting steroid synthesis involves the inhibition of enzymes responsible for their production, such as CYP17A1. The inhibition of CYP17A1 has been demonstrated to sensitize GBM cells to TMZ treatment [48, 50]. However, it is important to note that inhibiting CYP17A1 could potentially lead to side effects due to the role of this enzyme in normal steroid synthesis. These side effects could include fatigue, hypertension, fluid retention, and abnormal liver function tests [71]. Monitoring and appropriate management of these side effects are crucial when using CYP17A1 inhibitors in clinical settings. Another approach to suppress GBM involves the inhibition of the enzymes involved in arachidonate metabolism, such as 5-LOX and COX-2. Several enzyme inhibitors, including zileuton and celecoxib, have been developed and tested in preclinical studies. Inhibition of these enzymes has been shown to sensitize GBM cells to TMZ treatment and improve survival [44, 51, 72]. Importantly, it is critical to note that these inhibitors also carry potential side effects. Zileuton can cause liver toxicity [73], necessitating regular liver function tests. Celecoxib, on the other hand, carries a risk of gastrointestinal issues such as stomach pain, constipation, diarrhea, gas, heartburn, nausea, vomiting, and dizziness [74, 75]. Regular monitoring and patient education regarding the potential risks are crucial for safe administration of these drugs.

## Metabolomics for studying the dysregulation of lipid metabolism in GBM

### Metabolomics

The principle of metabolomics is to quantitatively measure and analyze a comprehensive set of small-molecule metabolites in biological systems, such as cells, tissues, or biofluids. Metabolomics aims to provide a snapshot of the metabolic state of the system under study and identify changes associated with biological processes, disease states, or environmental exposures in the metabolite profiles [76]. Metabolomics typically involves several steps, including sample collection, sample preparation, metabolite profiling, and data analysis. Biological samples, such as blood, urine, tissue, and cells, are collected from the system of interest. Samples may be collected from healthy individuals or patients with a disease or condition of interest. Samples are then processed to extract the metabolites and remove unwanted components, such as proteins and lipids, that may interfere with metabolite analysis. Sample preparation may involve various techniques, such as liquid-liquid extraction, solid-phase extraction, and derivatization. The extracted metabolites are then analyzed using high-throughput analytical techniques, such as gas chromatography-mass spectrometry (MS), liquid chromatography-MS, or nuclear magnetic resonance spectroscopy. These techniques provide quantitative measurements of the metabolites in samples. Raw metabolomic data obtained from metabolite profiling are processed and analyzed using statistical and bioinformatics tools to identify differences in metabolite profiles between different samples or conditions. Data analysis may involve various techniques, such as multivariate, pathway, and machine learning [76, 77]. Metabolomics is a powerful tool for quantifying and analyzing a comprehensive set of metabolites present in biological systems. By identifying changes in metabolite profiles, metabolomics can provide insights into the underlying biological processes and disease mechanisms, and may have applications in disease diagnosis, prognosis, and personalized medicine.

Metabolomics is a rapidly growing field that can revolutionize clinical diagnosis. By analyzing the metabolic profile of a biological sample, such as blood or urine, metabolomics can provide a comprehensive picture of the underlying biochemical changes associated with a particular disease or condition [77]. Importantly, metabolomics can be used to diagnose a wide range of diseases, including cancer, diabetes, cardiovascular diseases, and neurological disorders. Metabolomics can also be used to monitor disease progression and treatment response, and reveal new drug targets. However, several challenges must be overcome before metabolomics can be widely adopted for clinical diagnosis. These challenges include the standardization of sample collection and preparation, development of robust and reproducible analytical methods, and integration of metabolomic data with other clinical data. Despite these challenges, the potential benefits of metabolomics for clinical diagnosis are significant. Metabolomics can provide rapid and noninvasive diagnosis, identify new biomarkers, and enable personalized medicine by identifying subgroups of patients that might respond to a particular treatment. Therefore, metabolomics is worthy of development for clinical diagnosis.

### Targeted and untargeted metabolomics

Advanced MS technologies, such as time-of-flight (TOF), Fourier transform ion cyclotron resonance (FT-ICR), triple quadrupole (QqQ), Orbitrap, and quadrupole linear ion trap (Q-TRAP), have emerged as crucial tools in metabolomics research [78]. These techniques allow for the detailed characterization of diverse metabolites, such as amino acids, carbohydrates, vitamins, nucleotides, peptides, hormones, and fatty acids, covering a wide concentration range [79]. Moreover, MS equipment is highly compatible with separation techniques like liquid chromatography (LC), gas chromatography (GC), and capillary electrophoresis (CE), which minimize signal suppression, increase sensitivity of detection, and aid metabolite identification by providing a retention time identifier [80].

There are two main metabolomic approaches: targeted and untargeted. Untargeted metabolomics offers broad coverage of metabolite measurements, utilizing high-resolution full scan MS for quantitation and tandem MS for structure elucidation [81]. However, the detector in full scan mode can be easily saturated, thus limiting the detection range, quantitative accuracy, and sensitivity of untargeted metabolomics [82]. On the other hand, targeted metabolomics utilizes the multiple-reaction monitoring (MRM) mode to determine a predefined set of metabolites [83], offering high sensitivity and selectivity by isolating targets of interest and excluding bulk signals from the background matrix. While targeted metabolomics is considered the gold standard for metabolite quantification, its major limitation is the reduced metabolite coverage, often focusing on a small to moderate number of well-known compounds [84]. Recent researches have aimed to develop targeted metabolomics with broader metabolite coverage [85]. The single LC-QqQ MS or the “DITQM” method, can detect up to ~ 160 endogenous metabolites, covering a wide range of metabolic pathways [85, 86]. Other approaches, such as hybrid quadrupole-Orbitrap MS or Q-TRAP MS, have expanded the analysis to nearly 200 metabolites [87–89]. Some techniques have even combined QqQ instruments with multiple column separation systems to quantify more than 200 endogenous metabolites in plasma [90].

While these targeted metabolomics methods are useful, analyzing more than three hundred known metabolites in a given biological sample remains challenging. Additionally, the metabolic profiles used for constructing MRM transitions are often collected from one specific type of sample, limiting their applicability to different biological systems. Therefore, the advancement of methodologies that can handle larger volumes of metabolites and that are versatile across various biological systems is a critical future direction in the field of metabolomics.

### Recent advances in the application of metabolomics for studies on lipid metabolism in GBM

Metabolomic studies have revealed the lipid signatures associated with GBM malignancy, such as increased levels of lysophosphatidylcholine and phosphatidylcholine [91]. While these identified signatures offer promising insights into TMZ resistance, further rigorous research is required before they can be reliably used as potential biomarkers to guide treatment decisions for GBM. Metabolomic studies have also revealed the changes in lipid metabolism during the progression of GBM, including alterations in fatty acid metabolism and phospholipid biosynthesis. These changes may contribute to the development of GBM and may be targeted for therapeutic interventions. In recent studies, metabolomics was combined with other omics approaches, such as genomics and proteomics, to gain a more comprehensive understanding of lipid metabolism in GBM. Notably, the identification of key phosphorylation events, such as phosphorylated PTPN11 and PLCG1, underscores their potential role as molecular switches mediating the activation of oncogenic pathways. Furthermore, they may represent promising therapeutic targets, particularly in tumors with alterations in EGFR, TP53, and RB1, some of the most frequently mutated genes in GBM. Additionally, acetylation of histone H2B in classical-like and immune-low GBM is driven primarily by bromodomain proteins (BRDs), CREBBP, and EP300, potentially offering an epigenetic rationale for the aggressive behavior of these subtypes [54]. These integrated analyses have shed light on novel metabolic pathways and potential therapeutic targets [92]. Furthermore, advances in analytical techniques, such as imaging mass spectrometry, have enabled the spatial mapping of lipid metabolism in GBM, enabling the identification of lipid distributions and potential lipid-based targets for therapy [93]. Particularly, a comprehensive analysis of a GBM patient-derived xenograft model, which integrated mass spectrometry imaging, histology, magnetic resonance imaging, phosphoproteomics, and mRNA sequencing, showed that the distribution of the EGFR inhibitor erlotinib within intracranial tumors was insufficient to inhibit EGFR tyrosine kinase signaling, despite its promising in vitro efficacy [94]. Importantly, the development of computational tools for metabolomic data analysis, such as pathway enrichment analysis and machine learning algorithms, has facilitated the identification of altered metabolic pathways and potential biomarkers. These tools may aid the development of personalized therapies based on individual metabolic profiles [95]. Taken together, these recent advances in metabolomics have remarkably expanded our understanding of lipid metabolism in GBM and may pave the way for the development of novel therapeutic interventions and biomarkers for the improved diagnosis and treatment of this deadly disease.

## Limitations of developing anti-GBM drugs targeting lipid metabolism

### Clinical trials of drugs targeting lipid metabolism in GBM

Although drugs targeting lipid metabolism appear to exhibit therapeutic efficacy against GBM, such drugs have failed in clinical trials. Based on a phase 2 randomized, double-blind, placebo-controlled trial of newly diagnosed GBM [72], celecoxib, an inhibitor targeting COX2, did not result in significant improvement in overall survival or progression-free survival (PFS) in GBM patients. The phase 2 study described here evaluated the combination of TMZ, an established DNA damage-induced chemotherapy drug, with the angiogenesis inhibitors thalidomide and celecoxib, for treating newly diagnosed adult GBM patients who had stable disease following standard radiation therapy. The primary endpoint of the study was PFS at 4 months from study enrollment, and the secondary endpoint was overall survival (OS). The study involved 50 patients, with a median age of 54 years. From the study enrollment, the median PFS was 5.9 months, and 4-month PFS was 63%. The median OS was 12.6 months, and 1-year OS was 47%. Out of the 47 patients evaluable for the best response, none showed a complete response, while 5 (11%) showed a partial response. The majority of patients either had stable disease (47%) or progressive disease (34%). Interestingly, the study also tried to correlate the response to treatment with levels of certain angiogenic peptides. It was found that higher levels of vascular endothelial growth factor, a crucial factor in angiogenesis, showed a trend towards correlation with decreased OS and PFS, although it was not statistically significant. The findings of this study indicate that the addition of celecoxib and thalidomide to TMZ did not significantly improve the 4-month PFS, implying that the drug combination failed to meet the study’s primary endpoint. These results align with the broader context, wherein clinical trials of anti-GBM drugs targeting lipid metabolism, including celecoxib, have not shown substantial clinical benefits [72].

A Phase 2 study of atorvastatin, a competitive HMG-CoA reductase (HMGCR) inhibitor, combined with TMZ and radiation therapy for newly diagnosed GBM (NCT02029573) [96] is currently ongoing. The primary endpoint of this study was PFS at 6 months (PFS-6), a measure commonly used to assess the efficacy of cancer treatment. This clinical study enrolled 36 patients, with a median age of 52 years, and treated them with the atorvastatin combination therapy for a median duration of 6.2 months. At a median follow-up of 19 months, the PFS-6 rate was 66%, with a median PFS of 7.6 months. Notably, thrombocytopenia and neutropenia, which are high-grade hematological adverse events, were reported in 7% and 12% of patients, respectively. Unfortunately, despite the promising preclinical data, atorvastatin did not improve PFS-6 in this GBM patient population. This indicates that while statins may have some anti-cancer activity, their effectiveness in improving survival outcomes in GBM patients, at least as part of the tested combination regimen, appears limited. However, this study offered valuable insights into the prognostic role of low-density lipoprotein (LDL) levels in GBM patients. High baseline LDL levels were associated with worse survival, suggesting that LDL could serve as a potential biomarker for poor prognosis in GBM [96]. In summary, clinical trials of anti-GBM drugs targeting lipid metabolism have revealed no significant clinical benefits (e.g., celecoxib) [72]. More research is needed to identify optimal treatment regimens and better understand the mechanisms underlying these approaches.

### Limitation of drug discovery for GBM

Despite the potential of targeting lipid metabolism as a strategy for the development of anti-GBM drugs, several limitations and challenges must be addressed. First, most enzymes and signaling pathways involved in lipid metabolism are involved in normal cellular processes. Therefore, targeting these pathways may have off-target effects that are detrimental to normal cells and tissues. The lack of specificity can result in unwanted side effects and toxicity, thereby limiting the clinical utility of these drugs. Second, GBM cells are highly adaptive and develop resistance to chemotherapy and other targeted therapies. Aberrant activation of lipid metabolism may also lead to the development of resistance mechanisms in GBM cells, thereby limiting the long-term efficacy of these treatments. Third, lipid metabolism is intricately connected to other signaling pathways, such as cell growth and survival, inflammation, and immune system pathways. This complexity makes it difficult to develop drugs that target lipid metabolism without interfering with other essential cellular functions. Fourth, despite recent advances in our understanding of the role of lipid metabolism in GBM, many aspects of this process remain poorly understood. Limited knowledge of the underlying biology of lipid metabolism in GBM may hinder the development of effective therapies.

Fifth, many lipid metabolism-targeting drugs have poor solubility and bioavailability, which limit their ability to penetrate the BBB and reach GBM cells in the brain. Therefore, developing drugs with good drug delivery properties remains a major challenge in the development of lipid metabolism-targeting anti-GBM drugs [97, 98].

Although targeting lipid metabolism is a promising approach for developing anti-GBM drugs, several limitations and challenges must be addressed, including the lack of specificity, development of resistance, complex interplay with other signaling pathways, a limited understanding of lipid metabolism in GBM, and drug delivery. Addressing these challenges requires further research and development to generate effective and safe lipid metabolism-targeting drugs for the treatment of GBM.

## Conclusion

Advances in metabolomics and other omics approaches have markedly expanded our understanding of the complexity of lipid metabolism in GBM. Moreover, these insights may help identify new targets for drug discovery. The development of agents that target these pathways could lead to more effective therapies that overcome resistance to current treatments. As GBM is a complex disease with multiple genetic and metabolic alterations, combination therapies targeting multiple pathways may be more effective than single-agent therapies. Combining lipid metabolism-targeting agents with other therapies, such as radiation or immune checkpoint inhibitors, may enhance treatment efficacy. Importantly, personalized medical approaches based on individual metabolic profiles may play a significant role in the discovery of drugs targeting lipid metabolism in GBM. Metabolomics and other omics approaches can be used to identify patient-specific metabolic signatures, enabling treatments to be tailored accordingly, which may lead to more effective and targeted therapies.

Drug repurposing could be considered for the treatment of GBM. Existing drugs targeting lipid metabolic pathways may be repurposed for the treatment of GBM. For example, the antifungal drug, ketoconazole, inhibits GBM growth through targeting Hexokinase II (HK2)-mediated metabolism [99]. While we have made strides in understanding the role of lipid metabolism in GBM, there is still much to learn. The intricacies of lipid metabolism and its integration with other metabolic pathways in the context of GBM are still not entirely deciphered. Moreover, while repurposing existing drugs offers a promising approach, the safety and effectiveness of such treatments in GBM patients must be thoroughly evaluated.

In conclusion, it is apparent that while we have made significant progress in understanding the role of lipid metabolism in GBM and are heading towards promising therapeutic strategies, we are still at the beginning of this journey. More in-depth studies are warranted to fully understand the role of lipid metabolism in GBM. Nonetheless, the advancements in metabolomics and other omics technologies are equipping us with the necessary tools to continue our research on GBM, offering hope for improved outcomes in patients afflicted with this devastating disease.

## Data Availability

Not applicable.

## Abbreviations

GBM: Glioblastoma
TMZ: Temozolomide
BBB: Blood-brain barrier
FABP: Fatty acid binding protein
PUFAs: Polyunsaturated fatty acids
AA: Arachidonic acid
FASN: Fatty acid synthase
D6D: Delta-6-desaturase
SREBP: Sterol regulatory element-binding protein
ACC: Acetyl-CoA carboxylase
CPT1A: Carnitine palmitoyltransferase 1A
PPARγ: Peroxisome proliferator-activated receptor gamma
DHEA: Dehydroepiandrosterone
5-LOX: 5-lipoxygenase
COX-2: Cyclooxygenase-2
CYP: Cytochrome P450
PTGES: Prostaglandin E synthase
AR: Androgen receptor
MGMT: O^6^-methylguanine-DNA methyltransferase
ROS: Reactive oxygen species
PG: Prostaglandin
MS: Mass spectrometry
PFS: Progression-free survival
TOF: Time-of-flight
FT-ICR: Fourier transform ion cyclotron resonance
QqQ: Triple quadrupole
Q-TRAP: Orbitrap, and quadrupole linear ion trap
LC: Liquid chromatography
GC: Gas chromatography
CE: Capillary electrophoresis
MRM: Multiple-reaction monitoring
